# Antimicrobial susceptibility to last-resort antibiotics in carbapenemase-producing bacteria from Ukrainian patients

**DOI:** 10.1128/spectrum.01142-24

**Published:** 2024-09-24

**Authors:** Nelianne J. Verkaik, Cornelia C. H. Wielders, Hans den Boer, Diana Langerak, Marius Vogel, Sandra Witteveen, Angela de Haan, Jeroen Bos, Mireille van Westreenen, Daan W. Notermans, Antoni P. A. Hendrickx, on behalf of the participating laboratories, on behalf of the SWAB Working Group Surveillance of Antimicrobial Resistance

**Affiliations:** 1 Department of Medical Microbiology and Infectious Diseases, Erasmus University Medical Center, Rotterdam, the Netherlands; 2 Centre for Infectious Disease Control (CIb), National Institute for Public Health and the Environment (RIVM), Bilthoven, the Netherlands; JMI Laboratories, North Liberty, Iowa, USA

**Keywords:** carbapenemase-producing Enterobacterales, antimicrobial susceptibility testing, cefiderocol, *Acinetobacter baumannii*, multidrug-resistant microorganisms, *Pseudomonas aeruginosa*, ceftazidime–avibactam, ceftolozane–tazobactam, imipenem–relebactam, Ukraine, War

## Abstract

Since March 2022, an increase was observed in multidrug-resistant microorganisms (MDRO), associated with the hospital transfer of Ukrainian patients. The goal was to collect phenotypic susceptibility data and assess clinical implications. Carbapenemase-producing Enterobacterales (CPE, *n* = 96), *Pseudomonas aeruginosa* (CPPA, *n* = 20), and carbapenem-resistant *Acinetobacter baumannii–calcoaceticus* (CRAB, *n* = 6) from Ukrainian patients were obtained from March to December 2022 from the Dutch MDRO surveillance. Antimicrobial susceptibility testing was performed using broth microdilution (BMD) when available, fosfomycin agar dilution, disk diffusion (DD) for cefiderocol, and diverse gradient strips. All isolates were sequenced with Illumina next-generation sequencing. For meropenem, aminoglycosides, ceftazidime–avibactam, ceftolozane–tazobactam, and imipenem–relebactam, susceptibility rates were low (0%–30%), due to the high number of *bla*_NDM_-positive isolates (79/122; 65%). For cefiderocol, results depended on reading with or without microcolonies, applying EUCAST or CLSI breakpoints, and whether DD or BMD was used; e.g., for *Klebsiella pneumoniae*, 30%–97% were susceptible. For colistin, 103/111 (93%) non-intrinsically resistant CPE/CPPA/CRAB isolates were susceptible. For most CPE, a low minimal inhibitory concentration (MIC) of <0.5 mg/L was measured for tigecycline and ceftazidime–avibactam–aztreonam. For CPPA, cefiderocol tested susceptible in 65%–100% of isolates. For CRAB, ampicillin–sulbactam MICs were ≥128 mg/L; for sulbactam–durlobactam, 1–2 mg/L. Admission in a Ukrainian hospital in the last year was a risk factor for MDRO, and majority were screening isolates (79%). There is extensive phenotypic resistance to last-resort antibiotics in MDRO from Ukrainian patients. Interpretation of cefiderocol susceptibility results depends on several variables. When treating patients recently admitted in Ukraine, suspected for Gram-negative bacterial infection, this should be taken into consideration.

**IMPORTANCE:**

Since March 2022, multidrug-resistant microorganisms associated with Ukrainian patients have been detected in national surveillance systems of several European countries. We studied the phenotypic antimicrobial susceptibility to last-resort antibiotics of multidrug-resistant microorganisms from Ukrainian patients in the Netherlands and assessed clinical implications. Our research revealed that there was extensive phenotypic resistance to last-resort antibiotics. Healthcare professionals should be aware of multidrug-resistant microorganisms when treating patients recently admitted in Ukraine, suspected for Gram-negative bacterial infection.

## INTRODUCTION

Since March 2022, there has been an increase in multidrug-resistant microorganisms (MDRO) in Europe associated with the hospital transfer of patients with MDRO originating from Ukraine. Genomic surveillance revealed that the majority of the MDRO represented globally spread lineages that contain New Delhi metallo-β-lactamase (NDM) genes (1–3). The Dutch national laboratory pathogen surveillance monitors molecular characteristics of carbapenemase-producing Enterobacterales (CPE), carbapenemase-producing *Pseudomonas aeruginosa* (CPPA), and carbapenem-resistant *Acinetobacter baumannii-calcoaceticus* complex (CRAB). The surveillance contains genotypic data of isolates and restricted epidemiological data of the patients from whom isolates were obtained, but only limited phenotypic susceptibility data for the cultured bacteria (4, 5). Antimicrobial susceptibility data are important to optimize management of infections in hospitals to which Ukrainian patients are transferred. Therefore, the goal of this study was to collect phenotypic susceptibility and genomic data of MDRO from Ukrainian patients and discuss implications for clinical practice.

## MATERIALS AND METHODS

### Strain selection

MDRO isolates from Ukrainian patients obtained from March to December 2022 with confirmed carbapenemase production or increased MIC for meropenem were included (6). These strains were collected within the national surveillance coordinated by the National Institute for Public Health and the Environment (RIVM) (Table S1). Isolates were sent to Erasmus MC, Rotterdam, the Netherlands, for antimicrobial susceptibility testing (AST), after approval from participating laboratories (*n* = 20). The national surveillance allows one submitted isolate per patient per year, with the exception that consecutive isolates are allowed from the same patient if these are Enterobacterales species with other carbapenemase-encoding gene combinations when compared to the first isolate.

### Antimicrobial susceptibility testing of MDRO

Isolates were tested by broth microdilution (BMD) EUMDRXXF (Sensititre, Thermo Fisher Scientific) including amikacin, aztreonam, cefepime, ceftazidime/avibactam, ceftolozane/tazobactam, colistin, eravacycline, fosfomycin +glucose-6-phosphate, imipenem, imipenem/relebactam, meropenem, meropenem/vaborbactam, piperacillin/tazobactam, tigecycline, tobramycin, disk diffusion cefiderocol (Liofilchem), BMD cefiderocol (CompASP, Liofilchem), gradient strip test aztreonam/avibactam (Liofilchem), gradient strip test plazomicin (Liofilchem; for 80 isolates, due to availability), fosfomycin agar dilution (Liofilchem), Etest sulbactam/durlobactam (in case of CRAB, bioMérieux), and gradient strip ampicillin/sulbactam (in case of CRAB, Liofilchem). Mueller Hinton agar plates from BD were used. Gentamicin was tested by Vitek2 AST-N344 card (bioMérieux). Tests were performed according to manufacturer’s instructions. For microdilution, the reading of MIC was performed with the help of the Sensitre Vizion by two independent experienced technicians. Results were analyzed using European Committee on Antimicrobial Susceptibility Testing (EUCAST) clinical breakpoints (7). For cefiderocol, since breakpoints differ substantially between EUCAST and Clinical and Laboratory Standards Institute (CLSI; M100-ED33:2023 Performance Standards for Antimicrobial Susceptibility Testing, 33rd Edition), results were analyzed using both breakpoints.

### Whole-genome sequencing of MDRO

MDRO isolates included in this study were subjected to whole-genome-sequencing (WGS) using Illumina next-generation sequencing. DNA was extracted using the DNeasy Blood & Tissue Kit according to the manufacturer’s instructions (Qiagen, Hilden, Germany). DNA libraries were prepared using Nextera DNA Flex Library Prep kit (Illumina, San Diego, CA, USA), followed by paired-end sequencing (2 × 150 bp) on the Illumina NextSeq550 platform (Illumina, USA), according to the manufacturer’s protocols. Read quality analysis and *de novo* assembly was done with the Juno-assembly v2.0.2 pipeline (https://github.com/RIVM-bioinformatics/juno-assembly). Briefly, read quality assessment and filtering were done using FastQC and FastIP (7, 8). Genomes were assembled using SPAdes and curated with QUAST, CheckM, and Bbtools (9, 10). Sequence data were used for the identification of resistance genes using ResFinder (version 4.1.0) from the Center for Genomic Epidemiology (8). A threshold of 95% was used for identity and 60% for the minimum length for ResFinder. MLST and wgMLST were performed to determine genetic relatedness of CPE, CPPA, and CRAB isolates. For wgMLST, in-house schemes were used as described previously (9).

### Epidemiological data

Data that were collected included data on gender, age, information on the reason for sampling, hospitalization or visit/stay abroad, and whether the patient was colonized with or infected by MDRO. Data were collected by the Municipal Health Services for CPE as part of mandatory reporting and through national surveillance questionnaires by the laboratories for CPPA/CRAB.

## RESULTS

### Carbapenemase genes and genetic relatedness among MDRO from Ukrainian patients

**Fig 1:**
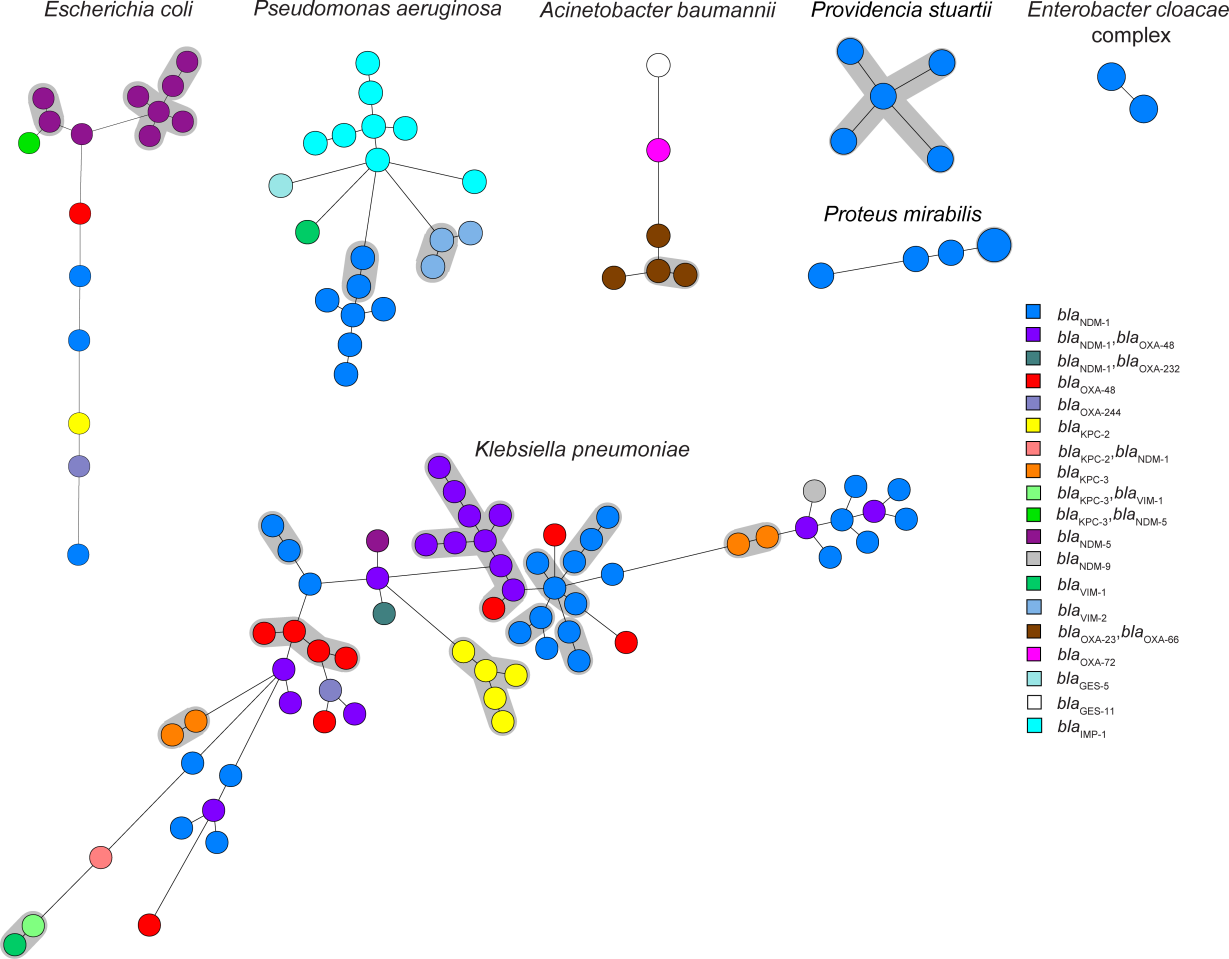
wgMLST analysis of MDRO from Ukrainian patients. Each circle represents an MDRO isolate per person, and the circle size indicates the number of isolates. Genetic clusters are indicated with gray halos. A genetic cluster of *K. pneumoniae*, *P. aeruginosa*, *E. coli*, *A. baumannii*, *P. mirabilis*, and *P. stuartii* isolates is defined as ≥2 isolates that differ by ≤20, ≤15, ≤25, ≤15, ≤15, and ≤15 alleles, respectively.

The 122 MDRO isolates concerned the following species: *n* = 66 *Klebsiella pneumoniae*, *n* = 20 *Pseudomonas aeruginosa*, *n* = 16 *Escherichia coli*, *n* = 6 *Acinetobacter baumannii–calcoaceticus* complex, *n* = 5 *Providencia stuartii*, *n* = 5 *Proteus mirabilis*, *n* = 2 *Enterobacter cloacae* complex, *n* = 1 *Proteus vulgaris*, and *n* = 1 *Citrobacter braakii* (Table 1; Table S1). *bla*_NDM_-like was the most frequently detected carbapenemase allele (79/122; 65%), followed by *bla*_OXA-48_-like (32/122; 26%), *bla*_KPC_-like alleles (13/122; 11%), *bla*_IMP_-like (8/122; 7%, all in *P. aeruginosa*), and *bla*_VIM_ (6/122; 5%, four in *P. aeruginosa*). The *bla*_GES_ gene was found in one *P. aeruginosa* isolate. The majority of *K. pneumoniae* isolates were from MLST ST147 (23/66; 35%), ST307 (11/66; 17%), ST395 (13/66; 20%), *E. coli* from ST46 (6/16; 38%), and *P. aeruginosa* isolates were mainly from ST773 and ST1047 (both STs 7/20; 35%). Other *bla*_OXA_ alleles different than *bla*_OXA-48_-like were *bla*_OXA-23_ and *bla*_OXA-66_ (in combination, *n* = 4), and *bla*_OXA-72_ (*n* = 1), all found in *A. baumannii* isolates with varying MLST STs. Twenty-nine of 122 isolates (24%) harbored combinations of two different carbapenemase genes or more, of which *bla*_NDM_ with *bla*_OXA-48_ was the most prevalent (20/29; 69%). In one CRAB isolate, no carbapenemase-allele was found. wgMLST analysis revealed 10 genetic clusters comprising *n* = 37 isolates for *K. pneumoniae*, two clusters (*n* = 4) for *P. aeruginosa*, two (*n* = 8) for *E. coli*, one (*n* = 2) for *A. baumannii*, one (*n* = 2) for *P. mirabilis*, and one (*n* = 5) for *P. stuartii*, while the remainder of the isolates were not genetically related (Fig. 1). A considerable proportion of the MDRO (58/122; 48%) appeared to be clonally spread among Ukrainian patients.

**TABLE 1:** Carbapenemase-encoding genes and sequence types of MDRO from Ukrainian patients

Species and carbapenemase allele	Total	MLST sequence type																																	
		10	13	14	15	23	39	46	69	147	231	235	307	361	392	395	405	446	512	654	744	773	1047	1100	2063	2592	2819	4981	5403	5859	14870	2063	546	New ST	Blank
***Acinetobacter baumannii***	6																							1	2		1					1	1		
*bla*OXA-23, *bla*OXA-66	4																								2							1	1		
*bla*OXA-72	1																										1								
Not found or incomplete	1																							1											
***Citrobacter braakii***	1																																	1	
*bla*NDM-1, *bla*OXA-48	1																																	1	
***Enterobacter cloacae complex***	2										2																								
*bla*NDM-1	2										2																								
***Escherichia coli***	16	1						6	1					2			2				1							1			2				
*bla*KPC-2	1																1																		
*bla*KPC-3, *bla*NDM-5	1													1																					
*bla*NDM-1	3								1												1							1							
*bla*NDM-5	9							6						1																	2				
*bla*OXA-244	1																1																		
*bla*OXA-48	1	1																																	
***Klebsiella pneumoniae***	66		1	2	1	4	5			23			11		1	13			2										1	2					
*bla*KPC-2	5						5																												
*bla*KPC-2, *bla*NDM-1	1				1																														
*bla*KPC-3	4												2						2																
*bla*KPC-3, *bla*VIM-1	1			1																															
*bla*NDM-1	25		1			3				12			6			3																			
*bla*NDM-1, *bla*OXA-232	1																													1					
*bla*NDM-1, *bla*OXA-48	16					1				9			2			3														1					
*bla*NDM-5	1															1																			
*bla*NDM-9	1												1																						
*bla*OXA-244	1															1																			
*bla*OXA-48	9									2					1	5													1						
*bla*VIM-1	1			1																															
***Proteus mirabilis***	5																																		5
*bla*NDM-1	5																																		5
***Proteus vulgaris***	1																																		1
*bla*NDM-1	1																																		1
***Providencia stuartii***	5																																		5
*bla*NDM-1	5																																		5
***Pseudomonas aeruginosa***	20											1						1		3		7	7			1									
*bla*GES-5	1											1																							
*bla*NDM-1	7																					7													
*bla*VIM-1	1																									1									
*bla*VIM-2	3																			3															
*bla*IMP-1	8																	1					7												
**Total**	122	1	1	2	1	4	5	6	1	23	2	1	11	2	1	13	2	1	2	3	1	7	7	1	2	1	1	1	1	2	2	1	1	1	11

### Antimicrobial susceptibility testing of MDRO from Ukrainian patients

**Fig 2:**
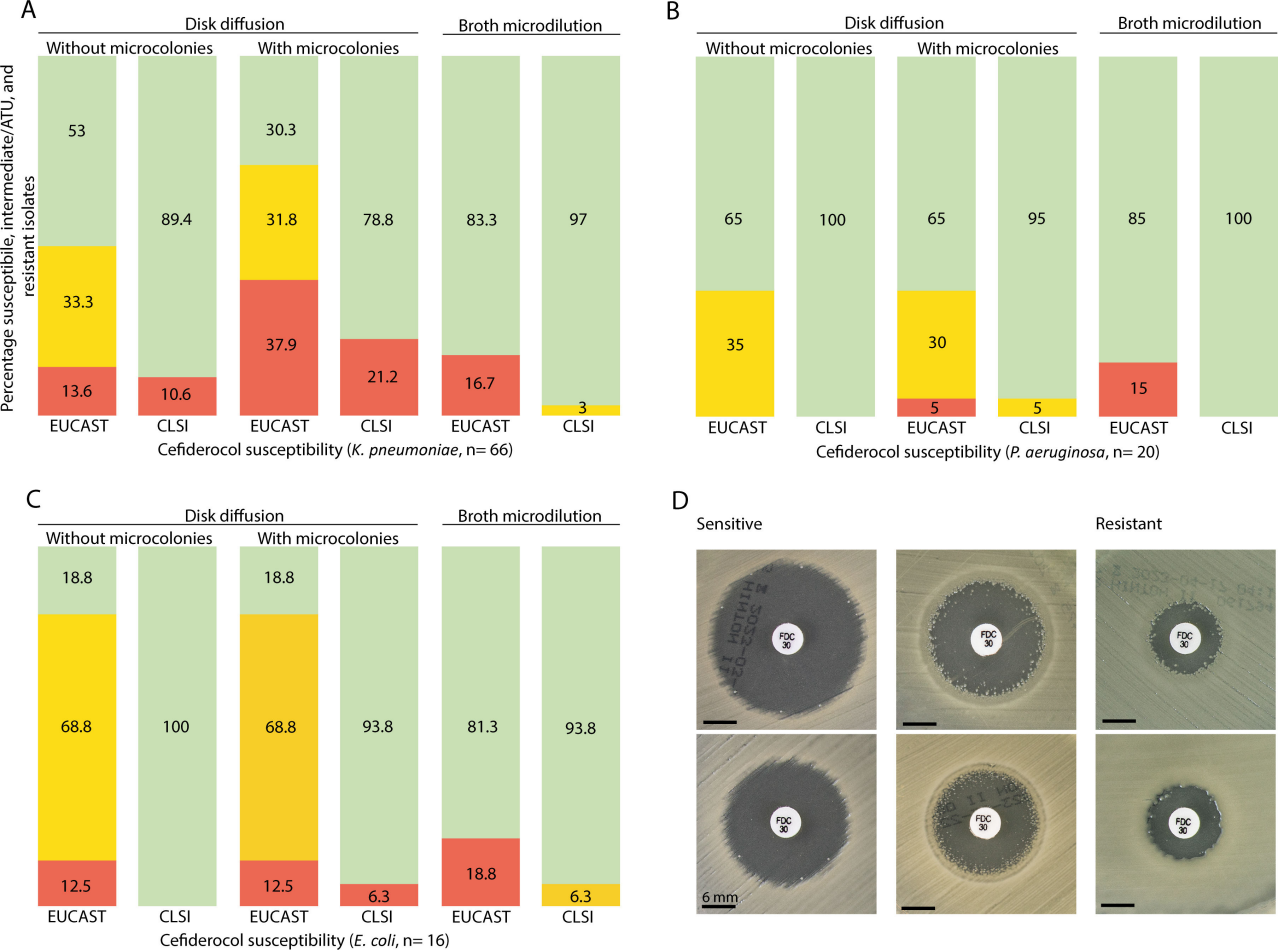
(A–C) Susceptibility of *K. pneumoniae*, *P. aeruginosa*, and *E. coli*, respectively, to cefiderocol. Green is the percentage of susceptible isolates, red is the percentage of resistant isolates, according to EUCAST and CLSI interpretation, respectively. Orange indicates the area of technical uncertainty (ATU) for EUCAST (18–21 mm for Enterobacterales, 14–21 mm for *P. aeruginosa*; 22 mm was regarded as susceptible) and indicates “I” for CLSI. (D) Mueller Hinton agar plates with susceptibility results for cefiderocol for six different *K. pneumoniae* isolates: two susceptible isolates, two resistant isolates (according to EUCAST and CLSI), and two isolates for which the interpretation was dependent on whether results were read with or without microcolonies.

Of all 122 MDRO isolates, 74% was resistant (R) for meropenem and >70% was R for aminoglycosides (amikacin, 84% R, tobramycine, 94% R, gentamicin, 72% R). The plazomycin MIC ranged from 0.25 to >256 mg/L (median 32 mg/L). AST of reserve antibiotics as defined by the WHO (10), including those which may still be active to *bla*_NDM-_positive MDRO, are depicted in Table 2. For cefiderocol, representative susceptibility results for *K. pneumoniae*, *P. aeruginosa*, and *E. coli* are depicted in Fig. 2. Different interpretation was obtained when results were read with or without microcolonies, and whether EUCAST or CLSI breakpoints were applied. Microcolonies were most frequently observed for *K. pneumoniae*. For CRAB, ampicillin–sulbactam MIC were 128 mg/L (*n* = 1) and >256 mg/L (*n* = 5). For sulbactam–durlobactam, MICs were 1 mg/L (*n* = 4) or 2 mg/L (*n* = 2).

**TABLE 2:** Number and proportion (%) of susceptible MDRO isolates according to EUCAST or, in case of cefiderocol, according to EUCAST and CLSI^*b*^

Species	Cefidercol DD (CLSI)	Cefiderocol DD (EUCAST)	Cefiderocol BMD (CLSI)	Cefiderocol BMD (EUCAST)	Cefolozane–tazobactam	Ceftazidime–avibactam	Ceftazidime–avibactam–aztreonam	Colistin	Fosfomycin	Imipenem–relebactam	Meropenem-vaborbactam	Tigecyclin
*K. pneumoniae* (*n* = 66)	Fig. 2	Fig. 2	64 (97%)	55 (83%)	0%	14 (21%)	*n* = 14 MIC ≤0.125, *n* = 29 MIC 0.25, *n* = 20 MIC 0.5, *n* = 2 MIC 1, *n* = 1 MIC 2	58 (88%)	21 (32%)	16 (24%)	25 (39%), *n* = 14 MIC 8	*n* = 46MIC ≤0.5, *n* = 15 MIC 1, *n* = 5 MIC >1
*P. aeruginosa* (*n* = 20)	Fig. 2	Fig. 2	20 (100%)	17 (85%)	1 (5%)	1 (5%)	*n* = 6 MIC 2, *n* = 5 MIC 4, *n* = 1 MIC 8, *n* = 5 MIC 16, *n* = 2 MIC 31, *n* = 1 MIC 64	20 (100%)	*n* = 1 MIC 8, *n* = 6MIC 16, *n* = 5 MIC 64, *n* = 8 MIC >256	1 (5%)	0 (0%)	NA
*E. coli* (*n* = 16)	Fig. 2	Fig. 2	15 (94%)	13 (81%)	1 (6%)	3 (19%)	*n* = 3 MIC <=0.125, *n* = 1 MIC 0.25, *n* = 2 MIC 0.5, *n* = 3 MIC 1, *n* = 1 MIC 2, *n* = 6 MIC ≥4	16 (100%)	16 (100%)	3 (19%)	6 (38%), *n* = 3 MIC 8	*n* = 12MIC ≤0.5, *n* = 1 MIC 1, *n* = 3 MIC >1
*A. baumannii* complex (*n* = 6)	4 (67%)^*a*^	4 (67%)	4 (67%)	4 (67%)	NA	NA	*n* = 1 MIC 32, *n* = 1 MIC 64, *n* = 4 MIC >256	6 (100%)	NA	0 (%)	0 (0%)	*n* = 6 MIC ≤0.5
*P. stuartii* (*n* = 5)	5 (100%)	5 (100%)	5 (100%)	5 (100%)	0 (0%)	0 (0%)	*n* = 4 MIC ≤0.125, *n* = 1 0.25	NA	3 (60%)	0 (0%)	3 (60%), *n* = 3 MIC 8	NA
*P. mirabilis* (*n* = 5)	5 (100%)	5 (100%)	5 (100%)	5 (100%)	0 (0%)	0 (0%)	*n* = 5 MIC ≤0.064	NA	0 (0%)	0 (0%)	4 (80%), *n* = 3 MIC 8	NA
*E. cloacae* (*n* = 2)	1 (50%)	0 (0%)	1 (50%)	1 (50%)	0 (0%)	0 (0%)	MIC 0.125 and 0.5	2 (100%)	2 (100%)	0 (0%)	0 (0%)	MIC ≤ 0.5 and 1
*P. vulgaris* (*n* = 1)	1 (100%)	1 (100%)	1 (100%)	1 (100%)	0 (0%)	0 (0%)	MIC < 0.016	NA	1 (100%)	0 (0%)	1 (100%)	NA
*C. braakii* (*n* = 1)	1 (100%)	1 (100%)	1 (100%)	1 (100%)	0 (0%)	0 (0%)	MIC 0.125	1 (100%)	1 (100%)	0 (0%)	1 (100%)	MIC 1

^*a*^ Two strains <14 mm according to CLSI, do not report.

^*b*^ DD, disk diffusion; BMD, broth microdilution; NA, no EUCAST breakpoint or activity known to be insufficient or intrinsic resistance. Occasionally, if there are no EUCAST breakpoints, MICs (mg/L) are presented. Since for tigecycline, there are breakpoints for *E. coli* only, therefore, MICs are presented. For fosfomycin, the breakpoint for intravenous application of 32 mg/L was applied.

### Epidemiology of Ukrainian patients

MDRO isolates were retrieved from 66 patients; 52 (79%) male and 14 female (21%) with a median age of 32 years (range 0–86 years; IQR 23–46). Most patients were sampled for screening purposes (79%) (Table 3; Table S2). Seventy percent of the patients had been hospitalized abroad within 2 months preceding sampling in the Netherlands. The hospitalizations abroad were reported to be in Ukraine (*n* = 42), Ukraine and Poland (*n* = 2), and Poland (*n* = 2). Nine patients (14%) were hospitalized in Ukraine >2 months but <1 year before sampling, while for 11 patients, only a visit/stay in Ukraine without hospitalization was reported. For 39 patients, one isolate was available, while for 27 patients, multiple isolates (different species and/or carbapenemase-encoding gene) were available: two isolates (*n* = 16 patients), three and four isolates (both *n* = 4 patients), five isolates (*n* = 2 patients), and for one patient, 13 separate isolates, leading to a total of 122 CPE, CPPA, and CRAB isolates from Ukrainian patients.

**TABLE 3:** Characteristics of 66 patients with respect to reason for sampling, hospitalization abroad, and whether there was an infection present or colonization

Characteristic	N (%)
Reason for sampling	
Screening	52 (79)
Diagnostic	12 (18)
Other	2 (3)
Hospitalization or visit/stay abroad	
<2 months before sampling for >24 h	46 (70)
>2 months but <1 year before sampling for > 24 h	9 (14)
Visit/stay <1 year before sampling without hospitalization	11 (17)
Colonization with or infected by MDRO	
Colonization	42 (64)
Wound infection	5 (8)
Urinary tract infection	2 (3)
Bacteremia/sepsis	1 (2)
Unknown/not reported	16 (24)

## DISCUSSION

We demonstrated extensive phenotypical antimicrobial resistance to last-resort antimicrobial drugs in carbapenemase-producing Gram-negative bacteria isolated from Ukrainian patients. This is most likely the result of the predominance of *bla*_NDM_-positive isolates, as a number of the new last resort beta-lactam/beta-lactamase combinations are not effective against metallo-beta-lactamases such as NDM. Although this was described before in war victims with nosocomial infections in Ukraine, this concerned a smaller number of last-resort antibiotics than the present study, and it was so far unknown for patients from Ukraine in the Netherlands (3). These data are required to optimize management of infections in hospitals to which patients are transferred (11).

The only antimicrobial drug that shows high susceptibility rates in CPE, for species that are not intrinsically resistant, CPPA, and CRAB, is colistin. Low susceptibility rates were found for aminoglycosides, ceftazidime–avibactam, ceftolozane–tazobactam, and imipenem–relebactam. For meropenem–vaborbactam, the MIC was occasionally 8 mg/L, which is exactly at the breakpoint. These data should be interpreted with caution since there were *bla*_NDM_-positive carrying isolates with this measured MIC, and a higher MIC was expected. For CPE, low MICs were measured for tigecycline and ceftazidime–avibactam–aztreonam. For CRAB, low MICs were observed for tigecycline and sulbactam–durlobactam.

For cefiderocol, the interpretation is influenced substantially by applying either EUCAST or CLSI breakpoints, whether or not microcolonies are taken into account, and whether DD or BMD was used. Microcolonies were present most frequently in *K. pneumoniae* (38/66 isolates). To illustrate the difference, when EUCAST breakpoints are applied, and microcolonies are ignored, 35/66 (53%) isolates are susceptible. However, if microcolonies are taken into account, another 15 isolates fall within the area of technical uncertainty (ATU) or become resistant, and only 20/66 (30%) remain susceptible. With CLSI breakpoints applied, the percentage of susceptible isolates is much higher (79%–89%). This is also the case if BMD is used (83%–97%).

This leads to a major dilemma in clinical practice. EUCAST recommends laboratories for cefiderocol DD with results inside the ATU, and as long as there is no alternative method to resolve interpretative uncertainties (e.g., MIC testing in the routine laboratory or assistance from a reference laboratory), to ignore the ATU and interpret using the zone diameter breakpoints in the breakpoint table (https://www.eucast.org/ast-of-bacteria/warnings). However, since treatment options for *bla*_NDM_-positive isolates are limited, and as long as there is difficulty in testing since results are markedly affected by iron concentration, discussion on what the breakpoint should be and more clinical data become available, in our opinion, cefiderocol should not be disregarded and reported resistant too quickly (12).

To assess the possible implications for clinical practice with respect to antibiotic policy, the limitations of this study need to be addressed first. The denominator misses as we do not know the number of Ukrainian patients who were admitted in the Netherlands, but tested negative for MDRO, since isolates are only sent to the RIVM if there was confirmed carbapenemase production or increased MIC for meropenem. Second, for patients with a visit/stay abroad without hospitalization <1 year ago (17%), details, such as underlying medical conditions or a potential unreported hospitalization, and denominator data, are lacking, making interpretation in this particular group of patients difficult. Third, most of the MDRO were screening isolates.

Thus, it is uncertain whether our findings of limited therapeutic options apply to all patients from Ukraine seen in Western hospitals. Therefore, we would advise to await for diagnostic culture results when possible. If not, for example, in case of sepsis, we suggest adding colistin to the local empirical antibiotic regimen of patients suspected for Gram-negative bacterial infection, if these patients were hospitalized in Ukraine in the last year, and no colonization data are available yet. If the bacterial species is known, therapy may be adjusted awaiting susceptibility results because of the potential toxicity of colistin, in particular, if the patient is suffering from impaired kidney function. For Ukrainian patients with infection caused by CPE, ceftazidime–avibactam plus aztreonam or high-dose tigecycline might be an option (13–15). However, these drugs are not approved for all indications by the United States Food and Drug Administration (FDA) and European Medicines Agency (EMA), clinical data are limited, and for ceftazidime–avibactam plus aztreonam, the effective concentration that reaches the site of infection is difficult to determine. Aztreonam–avibactam is currently under investigation, but not FDA or EMA approved yet (16). In addition, for infections caused by CPE, cefiderocol may be used, even in the presence of microcolonies given the uncertainties as mentioned before. Combination therapy may be advisable in patients with infections caused by *bla*_NDM_-positive isolates (17), although the IDSA guideline suggests monotherapy (18). Cefiderocol is EMA approved for infections caused by aerobic Gram-negative organisms in adults with limited treatment options. Clinical data on cefiderocol are also limited, though.

For CPPA, cefiderocol, and CRAB, either high-dose tigecycline (in combination), cefiderocol (in combination), or sulbactam–durlobactam can be considered, since for the latter drug, the proposed susceptibility breakpoint is 4 mg/L (19), which means all isolates can be considered susceptible. However, sulbactam–durlobactam is not yet approved for use in the European Union. For ampicillin–sulbactam, high MICs were measured in all CRAB isolates. This may still remain an effective treatment option, however, based on the potential for sulbactam to saturate altered penicillin-binding protein targets. A second or even a third agent in case of moderate-to-severe CRAB infections should be added (8).

The strengths of this study are that large-scale susceptibility data are systematically obtained according to standardized protocols, within an ISO15189-accredited laboratory for an important group of patients. Furthermore, detailed information is provided on cefiderocol susceptibility testing using different methods and breakpoints. In addition, we tested antimicrobial susceptibility to more last-resort antimicrobial drugs and presented more detailed information compared to the previous manuscript by Ljungquist et al. (3) Future research should focus on the best method to determine cefiderocol susceptibility, what the breakpoint should be, and how to interpret the results, taking clinical data, such as clinical outcome after treatment, into account.

In conclusion, there is extensive phenotypic antimicrobial resistance in carbapenemase-producing Gram-negative bacteria isolated from Ukrainian patients in the Netherlands. Risk factor for MDRO is admission in a hospital in Ukraine in the last year. For cefiderocol, interpretation of susceptibility results depends on a number of variables (microcolonies, breakpoint applied, method used). In case of treating Gram-negative infection in patients recently admitted in Ukraine, this should be taken into consideration.

## Data Availability

The Illumina (NGS) sequence data set generated and analyzed in this study is available in the Sequence Read Archive (SRA) with study accession number PRJNA903550.

## SUPPLEMENTAL MATERIAL

Table S1 spectrum.01142-24-s0001.xlsx Genotypic and phenotypic data of MDRO from Ukrainian patients.

Table S2 spectrum.01142-24-s0002.xlsx Epidemiological data of Ukrainian patients.

ASM does not own the copyrights to Supplemental Material that may be linked to, or accessed through, an article. The authors have granted ASM a non-exclusive, world-wide license to publish the Supplemental Material files. Please contact the corresponding author directly for reuse.
